# Rural/urban Background, Depression and Suicidal Ideation in Chinese College Students: A Cross-Sectional Study

**DOI:** 10.1371/journal.pone.0071313

**Published:** 2013-08-16

**Authors:** Heng Meng, Jian Li, Adrian Loerbroks, Jiao Wu, Hui Chen

**Affiliations:** 1 Department of Maternal and Child Health, School of Public Health, Tongji Medical College, Huazhong University of Science and Technology, Wuhan, China; 2 Tongji Center of Injury Prevention, Tongji Medical College, Huazhong University of Science and Technology, Wuhan, China; 3 Institute of Occupational and Social Medicine, Center for Health and Society, Faculty of Medicine, University of Düsseldorf, Düsseldorf, Germany; 4 Institute for Medical Sociology, Center for Health and Society, Faculty of Medicine, University of Düsseldorf, Düsseldorf, Germany; 5 Mannheim Institute of Public Health, Social and Preventive Medicine, Medical Faculty Mannheim, Heidelberg University, Mannheim, Germany; Chiba University Center for Forensic Mental Health, Japan

## Abstract

**Objectives:**

The objective of this study was to examine, first, the relationship of having a rural vs. urban background with suicidal ideation in Chinese college students, and second, whether a potential relationship was mediated by depression.

**Methods:**

A survey was conducted among 1,145 undergraduate students at a university in China. Suicidal ideation and depressive symptoms were measured by the revised Hopkins’ Symptom checklist (SCL-90-R). Associations between rural vs. urban background, depression and suicidal ideation were estimated by multivariable linear regression-based β coefficients, logistic regression-based odds ratios (ORs), and corresponding 95% confidence intervals (CIs). The magnitude of indirect effect and bias-corrected 95% CIs were obtained through bootstrap techniques.

**Results:**

Rural background was positively associated with depression, which was in turn associated with suicidal ideation. The OR for rural status and suicidal ideation equaled 2.15 (95% CI = 1.36–3.41). This OR was slightly, though significantly (p<0.05) attenuated by additional adjustment for depressive symptoms (OR = 1.99, 95% CI = 1.15–3.44).

**Conclusion:**

Having a rural background is a determinant of suicidal ideation in Chinese college students. Depression may only marginally mediate this association.

## Introduction

Suicide is one of the three leading causes of death among adolescents around the world [1], [2], [3]. In China, suicide ranks first among the cause of death observed in those aged 15–34 years, accounting for 19% of all deaths in this age group [1], [2], [3]. Suicidal ideation is defined as thoughts of harming or killing oneself. Suicidal ideation is an important predictor of suicide attempts and completed suicide and represents a significant marker for other mental health problems among youth. For these reasons, an understanding of its risk factors and protective factors is essential to inform strategies aiming at suicide prevention [4].

Young people between the ages of 18–30 years have the highest prevalence of reported suicidal ideation, and are more likely to have made a suicide plan, compared with older adults [5], [6], [7]. For young adults attending college, the risk of suicidal ideation is greater than for the same-age counterparts not attending college [8], [9]. The prevalence of suicidal ideation in the past year among college samples in China varied from 13.8% to 20.3% [10], [11], [12], [13], [14].

Suicidal ideation among college students has been shown to be associated with a variety of factors, which can be categorized into several domains including psychopathology, stressors, personality characteristics, and coping and problem solving skills [15]. There is a strong relationship between severity of depressive symptoms and suicidal ideation [16]. Previous studies also indicate that suicidal ideation and their correlates are influenced by social-cultural variables [17]. Overall, suicidal ideation levels have been found to be lower in less developed countries, particularly African, Asian, and Latin American countries [17], [18]. A study conducted in United States found that the prevalence of suicidal ideation was significantly lower among Chinese college students than among their American counterparts (34.3% vs. 42.2%) [19]. The lower prevalence of reported suicidal ideation rate among Chinese college students can be explained by several factors, including their homogeneous culture, less anomic life style, and lower levels of social competition [19]. Previous research has shown that risk factors for suicidal ideation and other suicidal outcomes identified in western societies may not be universally applicable. More extensive research is needed to explore the influence of social-culture factors on suicide, especially in countries in which suicide may be underreported or not well documented [17], [18].

A unique household registration system exists within the public administration structures of China, which is called Hukou. Hukou refers to the system of residency permits where household registration is required by law. Individuals are broadly categorized as “rural” or “urban” dwellers by the hereditary residency permits system. A factor which may be intimately linked to the prevalence of suicidal ideation in China is whether an individual comes from a rural regions (as opposed to urban region). Evidence indicated that a person’s rural background might be a marker for the experience of a range of socio-economic adversities as well as fewer social, economic and cultural benefits as compared to urban populations, which probably puts individuals with rural background at increased risk of suicidal behaviors [20]. People with a rural residency permit who migrate to work in urban areas are not eligible for equal education, employment, medical insurance, social welfare and other benefits that are available to urban dwellers. The transfer of residency permits from rural status to urban status is tightly controlled. For most of the rural children, passing the college entrance examination and becoming college students is the only chance to transfer to urban status. The current Hukou system is widely regarded as the cause of dramatic social, economic and educational differences between rural and urban areas in China [21].

The English and Chinese literature on correlates of suicidal ideation among college students in China has mainly focused on a few broad correlates, such as psychopathology and life stressors. Research on specific social-demographic determinants of self-reported suicidal ideation among college students is markedly sparse. To date, the potential relationship of rural vs. urban background with suicidal ideation among college students has not been explored, to our knowledge. Given that rural background is a marker of higher levels of socio-economic disadvantages and adversities in China and in view of the previously reported higher suicide rates in rural than in urban China, we hypothesize that college students from rural China will be more likely to report suicidal thoughts than students with an urban background. A second hypothesis of our study is that depression could partly mediate potential positive associations between a rural background and suicidal ideation. This hypothesis is in keeping with research documenting that rural residence status is significantly associated with depression in Chinese adolescents [22] and that depression represents a risk factor for suicidal ideation in China [23], [24].

## Methods

### Participants

Participants were recruited from a comprehensive university in Wuhan, Hubei Province, China. All of the students participating in an introductory course in Psychology were invited to participate in this study. The Introductory Psychology course is a general optional course available to undergraduate students of all specialties and grades in the university. A self-administered survey was conducted anonymously during normal class periods. The study was approved by the Ethics Committee of Tongji Medical College, Huazhgong University of Science and Technology. We obtained written informed consent from students aged over 18 years, and from the guardians on the behalf of the participants aged less than 18 years. 1170 of the 1300 students participating in the course returned the questionnaire voluntarily, which equals a response rate of 90.0%. A total of 1145 questionnaires without missing values were included for current analyses.

### Measures

The questionnaire assessed socio-demographic characteristics, recent negative life events and health-related lifestyle behaviors. Socio-demographic variables included age, gender, grade, and household background. Household background was defined as the type of household registered residency before entering college, which was recorded in two categories, rural residency and urban residency, in accordance with the Household Registration Management System in China.

Recent negative life events included physical health (“Have you had a serious illness during the past four weeks?”), academic performance (“Have you had poor academic performance during the past four weeks?”), financial problems (“Have you had a financial problem during the past four weeks?”), and interpersonal relationship conflicts (“Have you been involved in interpersonal conflicts during the past four weeks?”). Lifestyle behaviors included cigarette smoking and alcohol consumption. Cigarette smoking was assessed by the question of whether or not the student had used at least one tobacco product a week in the past four weeks. Drinking was measured by whether the student had used alcohol at least once a week in the past four weeks.

Suicidal ideation and depression were measured by items derived from the revised Hopkins’ Symptom checklist (SCL-90-R). The SCL-90-R is a self-report instrument measuring psychological symptoms and psychopathologic features. The items are rated on a five-point Likert scale of distress in the previous week: “not at all” (0), “a little bit” (1), “moderately” (2), to “quite a lot” (3) and “extremely often” (4). This instrument has been used extensively to measure a variety of mental disorders in clinical and non-clinical populations [25]. We used an available Chinese version of SCL-90-R with satisfactory reliability and validity [26]. There are two items of SCL-90-R asking about suicide thoughts, i.e. item 15 (“In the previous week, how much were you distressed by thoughts of ending your life?”) and item 59 (“In the previous week, how much were you distressed by thoughts of death or dying?”). Suicidal ideation was measured by these two items simultaneously. Students were considered positive on suicidal ideation if they had a score of 3 or 4 on both items (“quite a lot/extremely often”). Measurement of suicidal ideation based on the SCL-90-R items has been successfully used in previous studies and been confirmed to be significantly related to suicide attempts in clinical samples of people diagnosed with an eating disorder [27], [28], [29]. The cut-off we used in this study is higher than the cut-offs used in the above papers, hence suicidal ideation may considered as severe suicide ideation. We assessed depression using the SCL-90-R depression subscale with continuous score ranged from 0 to 4, high score indicating high level of depression.

### Data Analysis

**Figure 1:**
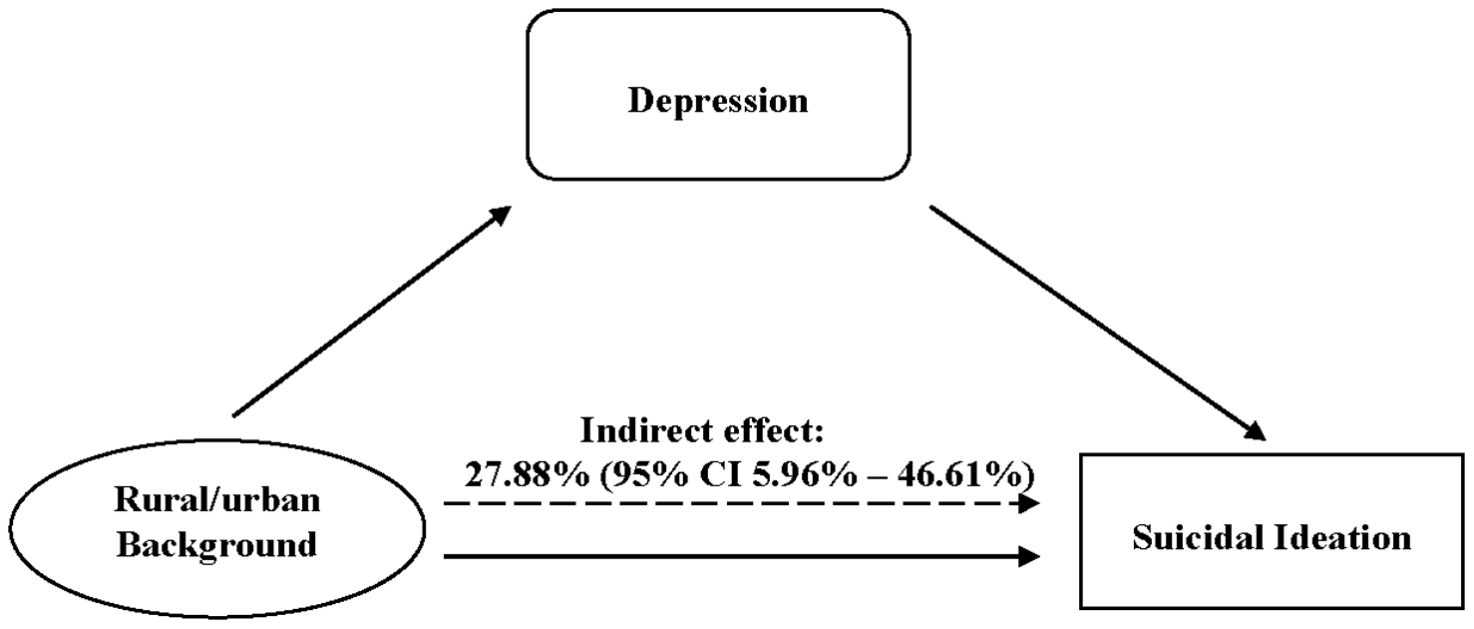
Model of the mediating role of depression on the association between rural/urban background and suicidal ideation.

Following descriptive statistics we applied Student’s *t*-test (for continuous variables) or Chi-square test (for categorical variables) to compare the differences between the two groups of students with urban or rural status. In order to examine the associations between rural/urban background and suicidal ideation mediated by depression, the 4-step mediation analysis was applied according to the recommendation by Baron and Kenny [30]. Therefore, we ran the following statistical regression models to explore the hypothesized associations depicted in Figure 1: first, we examined the potential association of rural-urban status with depression using linear regression; second, we assessed whether depression was positively linked to suicidal ideation using logistic regression; third, we investigated whether rural-urban status is related to suicidal ideation using logistic regression; and, fourth, whether this association is attenuated towards the null value of 1.0 after adjustment for depression using logistic regression. β coefficient and odd ratios (ORs) with 95% confidence intervals (CIs) served as the measures of association, adjusted for the potentially confounding effects of age, gender, grade, cigarette smoking, alcohol drinking, and four negative life events. The size of indirect effect and bias-corrected 95% CI were obtained through bootstrap techniques with 1000 replications [31]. All analyses were performed using SAS 9.2.

## Results

In total, 702 women and 443 men were included in this study. The age ranged from 16 to 33 years with a mean of 21.7 years. Overall, about one third of students came from a rural region. Characteristics of students by urban and rural background are summarized in Table 1. Compared with students from urban region, those who came from rural region had a significantly higher prevalence rate of suicide ideation (15.24% vs. 9.21%, p<0.01), and a significantly higher depression mean score (0.71 vs. 0.58, p<0.001).

**Table 1:** Characteristics of study sample (N = 1145).

Variable		Urban background (N = 771)N (%)	Rural background (N = 374)N (%)	*P*
Age (years, mean ± SD)		21.61±1.66	21.82±1.73	0.0456
Gender	Men	250 (32.43)	193 (51.60)	<0.0001
	Women	521 (67.57)	181 (48.40)	
Grade	Freshman	235 (30.48)	90 (24.06)	0.0018
	Sophomore	255 (33.07)	107 (28.61)	
	Junior+Senior	281 (36.45)	177 (47.33)	
Cigarette smoking	No	693 (89.88)	318 (85.03)	0.0165
	Yes	78 (10.12)	56 (14.97)	
Alcohol drinking	No	389 (50.45)	166 (44.39)	0.0540
	Yes	382 (49.55)	208 (55.61)	
Physical illness	No	748 (97.02)	358 (95.72)	0.2572
	Yes	23 (2.98)	16 (4.28)	
Poor academic performance	No	721 (93.51)	350 (93.58)	0.9650
	Yes	50 (6.49)	24 (6.42)	
Financial problems	No	465 (60.31)	302 (80.75)	<0.0001
	Yes	306 (39.69)	72 (19.25)	
Interpersonal conflicts	No	764 (99.09)	368 (98.40)	0.2969
	Yes	7 (0.91)	6 (1.60)	
Depression (mean ± SD)		0.58±0.57	0.71±0.63	0.0005
Suicide ideation	No	700 (90.79)	317 (84.76)	0.0024
	Yes	71 (9.21)	57 (15.24)	

Differences were determined by Student’s *t*-test or Chi-square test.

As shown in Table 2, after adjustment for age, gender, grade, cigarette smoking, alcohol drinking, and negative life events, we observed positive associations between rural- status (vs. urban status) and depression (β = 0.19, 95% CI = 0.05–0.33), and between depression and suicidal ideation (OR = 4.36, 95% CI = 3.45–5.50). Rural status was significantly associated with suicidal ideation (OR = 2.15, 95% CI = 1.36–3.41). After additional adjustment for depression, the OR was attenuated to 1.99 (95% CI = 1.15–3.44). As illustrated in Figure 1, the size of indirect effect was 27.88% (95% CI = 5.96%–46.61%) which is significant.

**Table 2:** Associations among rural-urban background, depression, and suicidal ideation.

Independent variables		Dependent variables
		**Depression (Linear regression #, β and 95% CI)**
Rural-urban status	Urban background	0
	Rural background	0.19 (0.05–0.33) **
		**Suicidal ideation (Logistic regression #, OR and 95% CI)**
Depression	Continuous	4.36 (3.45–5.50) ***
		**Suicidal ideation (Logistic regression #, OR and 95% CI)**
Rural-urban status	Urban background	1
	Rural background	2.15 (1.36–3.41) **
		**Suicidal ideation (Logistic regression ##, OR and 95% CI)**
Rural-urban status	Urban background	1
	Rural background	1.99 (1.15–3.44) *

* *P*<0.05,

** *P*<0.01,

*** *P*<0.001.

# Adjusted for age, gender, grade, cigarette smoking, alcohol drinking, and negative life events.

## Additionally adjusted for depression.

## Discussion

The key findings from our study are, first, that a rural background is a determinant of suicidal ideation in Chinese college students, and second, that depression may mediate this association to a limited extent. The findings of a positive association between rural background and suicidal ideation may be due to the fact that students from rural areas are more likely to have had poor family environments and are considered as having a lower social status, generally showing disadvantages [32]. The early negative life event of poor general family environment has a mild impact on suicidal behaviour, and a stronger impact on cognitive deficits, which in turn may have a strong impact on suicidal behaviour in college students [33].

The prevalence of suicidal ideation observed in this sample of college students was in line with the prevalence reported in previous studies from China and from western countries [10], [30], [31]. Further, students with a rural background exhibited a higher prevalence of suicidal ideation than those with urban background. This may possibly be ascribed to a higher rate of psychological distress among students from rural households and to their social disadvantages [32]. A positive association between rural versus urban background and depression was also demonstrated in this study: students from rural areas were more likely to have depression than those with urban backgrounds. Previous studies found that rural residency was associated with depression not only in adolescents, but also in individuals aged 18 and older in rural China. Within-country rural-to-urban migration in China has been widely confirmed to affect the mental health status of children and adolescents [34], [35], [36]. Social factors such as socioeconomic status of the family were negatively associated with depression and anxiety symptoms among medical students [37].

Even though suicidal ideation may occur in a range of disorders other than depression, and occasionally occurs in people without any psychiatric diagnosis [38], plenty of existing research suggested that depression appears to be a precondition for the presence of suicidal ideation [39], [40], [41]. There is a prominent association between depressive symptoms and suicidal ideations in university undergraduates [42], [43]. Those students with the most severe symptoms of depression were more likely to experience current suicidal ideation and conversely those students with suicidal ideation had worse symptoms of depression. Since suicidal ideation is among the diagnostic criteria for depression, presence of suicidal ideation in itself will necessarily increase the number of depressive symptoms [40], [41]. Consistent with results previously reported, we found that depression was strongly associated with suicidal ideation with the adjusted OR 4.36, suggesting that each 1-SD increase of the depression score was associated with roughly 4-fold elevated odds among students to report suicidal ideation.

Suicide in China has generally been understood not only as a consequence of mental illness, but also more frequently as a response to social inequalities [44]. The main goal of the current study was to better understand the relationship between rural/urban background and suicidal ideation and to examine to what extent depression may mediate this relationship. Social factors have been related to depression and suicidal ideation; however, these relationships are often more complex than main effect models may indicate. The findings from this study suggest that depression exerts main effects on suicidal ideation, whereas the mediating effect of depression between rural-urban background and suicidal ideation is only marginal. A cross-cultural study in the USA and China found that, a theoretical model was applied which explained the American college students’ suicidal ideation better than the ideation of Chinese students. While 41.5% of the variance of suicidal ideation was explained by the latent constructs of “depression”, “pro-suicide attitudes”, and “suicidal ideation” in the American sample, only 17.7% of the variance was accounted for in the Chinese sample [19]. This implies that suicidal ideation is explained to a lesser extent by depression in Chinese students as compared to American students. A number of alternative factors might better explain Chinese college students’ suicidal behaviours, such as lower social integration and higher discrimination [9], [45], [46]. Obviously, the mechanism calls for further research.

Our study is constrained by a number of limitations. Most importantly, we used a cross-sectional design which does not allow elucidating of whether a rural background is a potential cause of subsequent suicidal ideation. Our sample of students attending an Introductory Psychology class may not be representative for other student samples in China. The level of alcohol drinking was simply classified into “drinkers” and “non-drinkers”, that any possible effects of drinking and suicidal thoughts may not be apparent with the current classification. While we were able to account for a number of important confounders, residual or unmeasured confounding cannot be ruled out. The high response rate of our study minimized potential selection bias.

In conclusion, the results of this study highlight the role of a rural background in suicidal ideation in Chinese college students. These findings may enhance our understanding of variations in depression and the role of social factors in suicidal ideation, as well as contribute useful information to the prevention of suicidal behaviors in college students. The socio-demographic factors that have been found to be associated with suicidal ideation require attention in the contexts of screening of persons at high risk, suicide prevention programmes, and mental health promotion in colleges and universities. Future studies of risk factors and preventive factors for suicidal behaviours in Chinese college students should distinguish between rural and urban students in order to better understand how to decrease the apparent greater risk of suicidal behaviors in students from rural backgrounds.
